# A Rare Case of Gastropleural Fistula During Chemotherapy for Metastatic Colorectal Cancer

**DOI:** 10.7759/cureus.79937

**Published:** 2025-03-02

**Authors:** Yoko Shono, Akinori Sekioka, Tetsuo Ito, Kunihiko Tsuboi, Shuichi Ota

**Affiliations:** 1 Gastroenterological Surgery, Osaka Saiseikai-Noe Hospital, Osaka, JPN

**Keywords:** anti-vascular endothelial growth factor, chemotherapy, colorectal cancer, gastropleural fistula, peritoneal metastases

## Abstract

Gastropleural fistula (GPF) is a rare and life-threatening condition, often associated with peptic ulcer disease, trauma, abscess, malignancies, prior surgeries, or chemoradiation therapy. Its rarity, diverse clinical presentations, and the general condition of affected patients make GPF challenging to diagnose and treat. We present a case of GPF occurring during chemotherapy with anti-vascular endothelial growth factor (anti-VEGF) therapy for stage IV colon cancer with multiple peritoneal metastases. The patient underwent surgical repair of the fistula four weeks after the last administration of anti-VEGF therapy. Postoperative recovery was uneventful, and the patient successfully resumed oral intake. To the best of our knowledge, this is the first reported case of GPF in the context of stage IV colon cancer. This case highlights the importance of selecting an appropriate timing for surgical intervention, taking into account the patient’s overall condition and treatment history.

## Introduction

Gastropleural fistula (GPF) is an abnormal communication between the stomach and the pleural cavity. It is most commonly reported as a consequence of peptic ulcer disease, trauma, subdiaphragmatic abscess, empyema, malignancies, surgical procedures, or chemoradiation therapy [1-6]. The diagnosis and management of GPF are challenging due to its diverse etiologies and clinical manifestations. Radiological examinations, including chest X-ray, computed tomography (CT), or upper gastrointestinal (GI) series, are critical for confirming the diagnosis [7-9]. Although most patients initially undergo conservative treatment, surgical intervention is often necessary. Procedures such as laparotomy, thoracotomy, or endoscopic approaches may be employed. However, determining the optimal timing for surgery is particularly delicate [5-10].

GPF associated with cancer or chemotherapy is rare. Notably, there have been no previous reports of GPF associated with peritoneal metastases from colorectal cancer [2-6,8,10-14].

Here, we present a rare case of GPF that occurred during treatment for stage IV colon cancer with multiple peritoneal metastases.

## Case presentation

**Figure 1:**
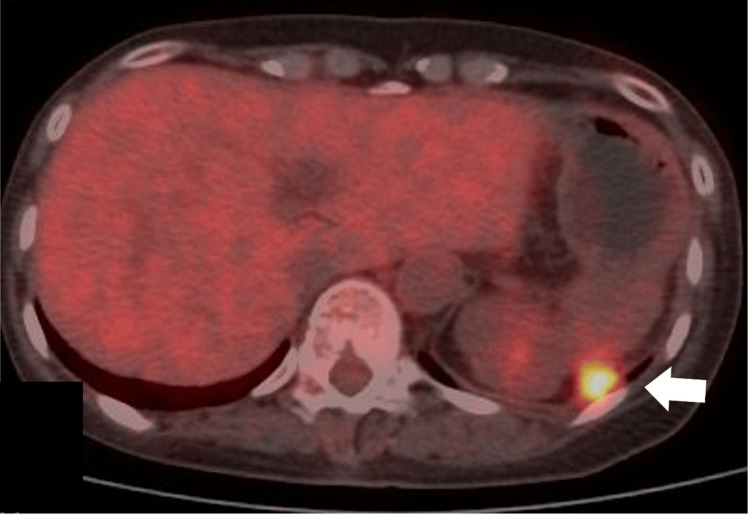
Positron emission tomography combined with computed tomography demonstrating a peritoneal metastasis at the left subdiaphragm (arrow)

A 61-year-old female (159cm, 51.2kg, body mass index 20.2kg/m^2^) with an only history of stage IV descending colon cancer presented with left-sided back pain and mild dyspnea lasting for a few days. The patient’s medical history included laparoscopic left hemicolectomy for the primary tumor 2.5 years ago, laparoscopic bilateral salphingo-oophorectomy for heterochronous ovarian metastases 1.5 years ago, and open distal pancreatectomy with splenectomy for localized peritoneal metastasis one year ago. Intraoperative findings during each procedure revealed no other metastasis than localized metastasis and no injury. There were mild adhesions only around the spleen during open distal pancreatectomy with splenectomy. Histological examinations of all these specimens revealed poorly differentiated adenocarcinoma, consistent with primary descending colon cancer. Following the last surgical procedure, the patient underwent six cycles of FOLFOX+BEV (fluorouracil, levofolinate calcium, oxaliplatin, and bevacizumab) and one cycle of FOLFIRI+RAM (fluorouracil, levofolinate calcium, irinotecan, and ramucirumab) for multiple peritoneal metastases located in the left subdiaphragm and the Douglas pouch over the past four months (Figure 1).

Five days before the presentation, the patient underwent the last cycle of FOLFIRI+RAM. On examination, her vital signs included a blood pressure of 169/92 mmHg, heart rate of 112 bpm, body temperature of 36.1℃, and peripheral oxygen saturation of 92% on room air. Laboratory findings demonstrated significantly elevated inflammatory markers, mild liver and renal dysfunction, and poor nutritional status on admission. The changes over time in the laboratory test results are shown in Table 1. 

**Table 1:** Laboratory data WBC: white blood cell, RBC: red blood cell, HGB: hemoglobin, HCT: hematocrit, CRP: C-reactive protein, PCT: procalcitonin, AST: serum aspartate aminotransferase, ALT: alanine aminotransferase, BUN: blood urea nitrogen, Na: sodium, K: potassium, Cl: chlorine, CEA: carcinoembryonic antigen, CA19-9: carbohydrate antigen 19-9, N/A: not applicable, POD: postoperative day

Test (unit)	Admission	Preoperative	Postoperative (POD 7)	Discharge (POD 51)	Reference range
WBC (x10^3^/μL)	27.0	14.9	13.3	10.6	3.5-8.0
RBC (×10^6^/μL)	4.28	3.05	3.41	3.54	3.8-4.8
HGB (g/dL)	14.7	10.3	11.0	11.1	11.3-14.9
HCT (%)	44.5	33.0	34.7	34.6	36.0-47.0
Platelets (x10^3^/μL)	281	584	569	628	120-400
CRP (mg/dL)	60.8	8.9	4.9	4.9	<0.3
PCT (ng/mL)	8.45	0.21	0.25	0.15	<0.05
β-D-glucan (pg/mL)	30.3	28.0	36.6	37.8	<20
Total protein (g/dL)	6.3	5.2	6.1	6.2	6.5-8.2
Serum albumin (g/dL)	3.1	1.8	2.7	2.8	3.7-5.5
Prealbumin (mg/dL)	6.6	11.0	22.6	15.3	22-40
AST (IU/L)	25	28	18	27	10-40
ALT (IU/L)	33	17	15	14	5-45
BUN (mg/dL)	23.5	9.9	10.7	18.4	8.0-20.0
Serum creatinine (mg/dL)	1.02	0.54	0.49	0.77	0.5-0.8
Na (mEq/L)	139	143	149	134	136-148
K (mEq/L)	4.3	4.9	4.5	4.5	3.6-5.0
Cl (mEq/L)	98	107	110	95	97-108
CEA (ng/mL)	21.1	N/A	N/A	239.0	<5.0
CA19-9 (U/mL)	95.8	N/A	N/A	239.0	<37.0

**Figure 2:**
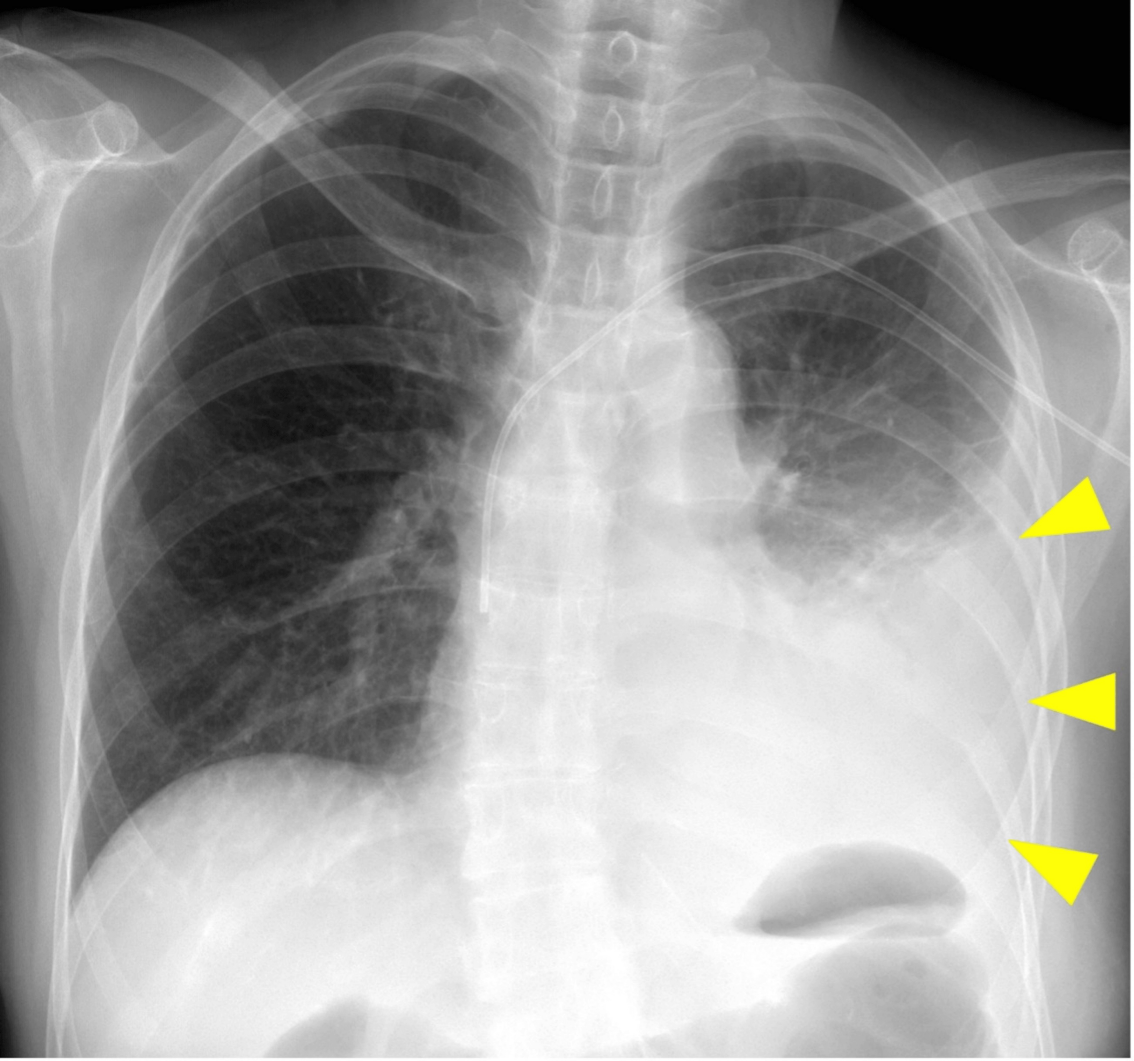
Chest X-ray revealing a large left pleural effusion (arrowheads)

A chest X-ray revealed left pleural effusion (Figure 2).

**Figure 3:**
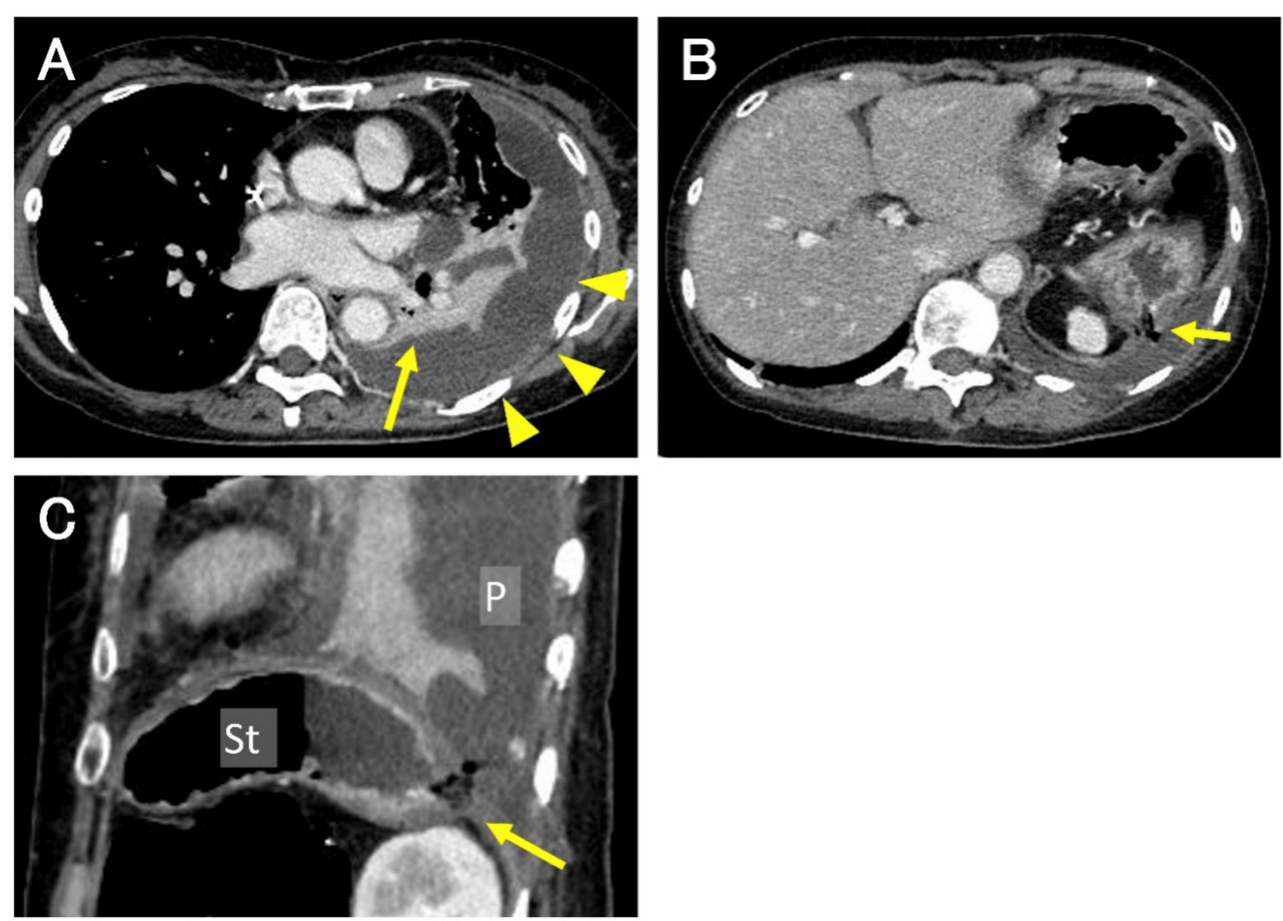
Contrast-enhanced computed tomography (A) Axial view of contrast-enhanced computed tomography (CECT) showing a significant fluid collection in the left pleural cavity (arrowheads) and left basilar atelectasis (arrow) (B) Axial view of CECT suggesting a fistula between the stomach and the left pleural cavity, corresponding to the location of the peritoneal metastasis at the left subdiaphragm (arrow) (C) Sagittal view of CECT demonstrating the fistula (arrow) St: stomach, P: pleural cavity

Contrast-enhanced CT demonstrated a left empyema containing gas, left basilar atelectasis (Figure 3A), and a fistula extending from the gastric fundus to the left pleural cavity through the peritoneal metastasis at the left subdiaphragmatic lesion (Figure 3B, 3C).

**Figure 4:**
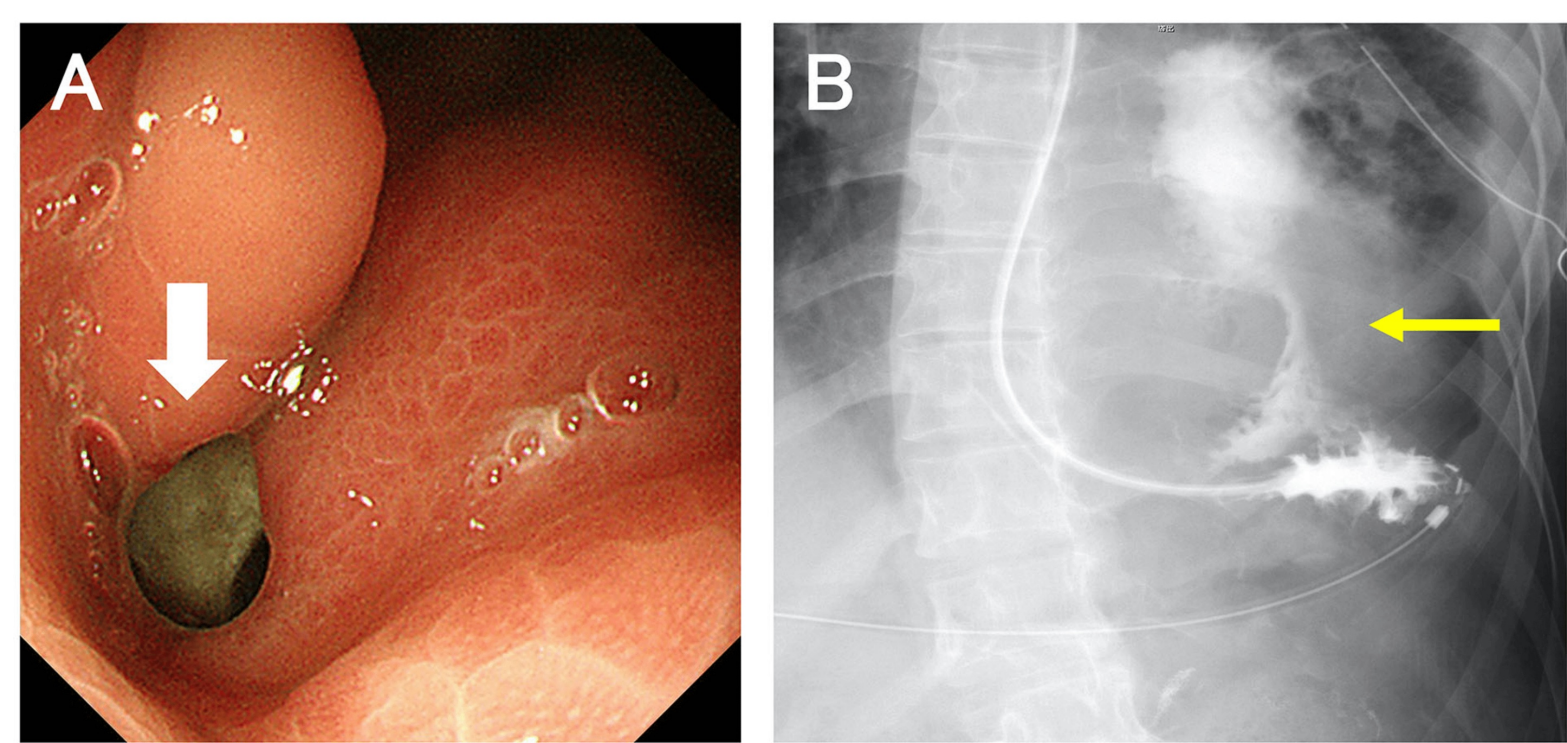
Esophagogastroduodenoscopy and upper gastrointestinal series (A) Esophagogastroduodenoscopy revealing a defect in the gastric fundus communicating with the pleural cavity (arrow) (B) Upper gastrointestinal series via nasojejunal tube demonstrating contrast leakage (arrow) from the stomach into the left pleural cavity

A chest tube was inserted, yielding cloudy yellow drainage without air leakage. Cytological analysis of the drainage fluid was negative for malignancy, while bacterial cultures identified *Streptococcus oralis* and *Candida albicans*. Esophagogastroduodenoscopy revealed a 1.0 cm fistula in the gastric fundus communicating with the left pleural cavity (Figure 4A). Based on these findings, the patient was diagnosed with GPF as the cause of the left empyema.

Conservative management was initiated, including broad-spectrum antibiotics (tazobactam-piperacillin), antifungal therapy (micafungin), proton pump inhibitors, parental nutrition (PN), and enteral nutrition (EN) via a nasojejunal feeding tube. However, after four weeks, the left empyema persisted, as confirmed by a GI series (Figure 4B).

The patient consented to palliative surgery for oral intake, and surgical repair of the fistula was performed.

**Figure 5:**
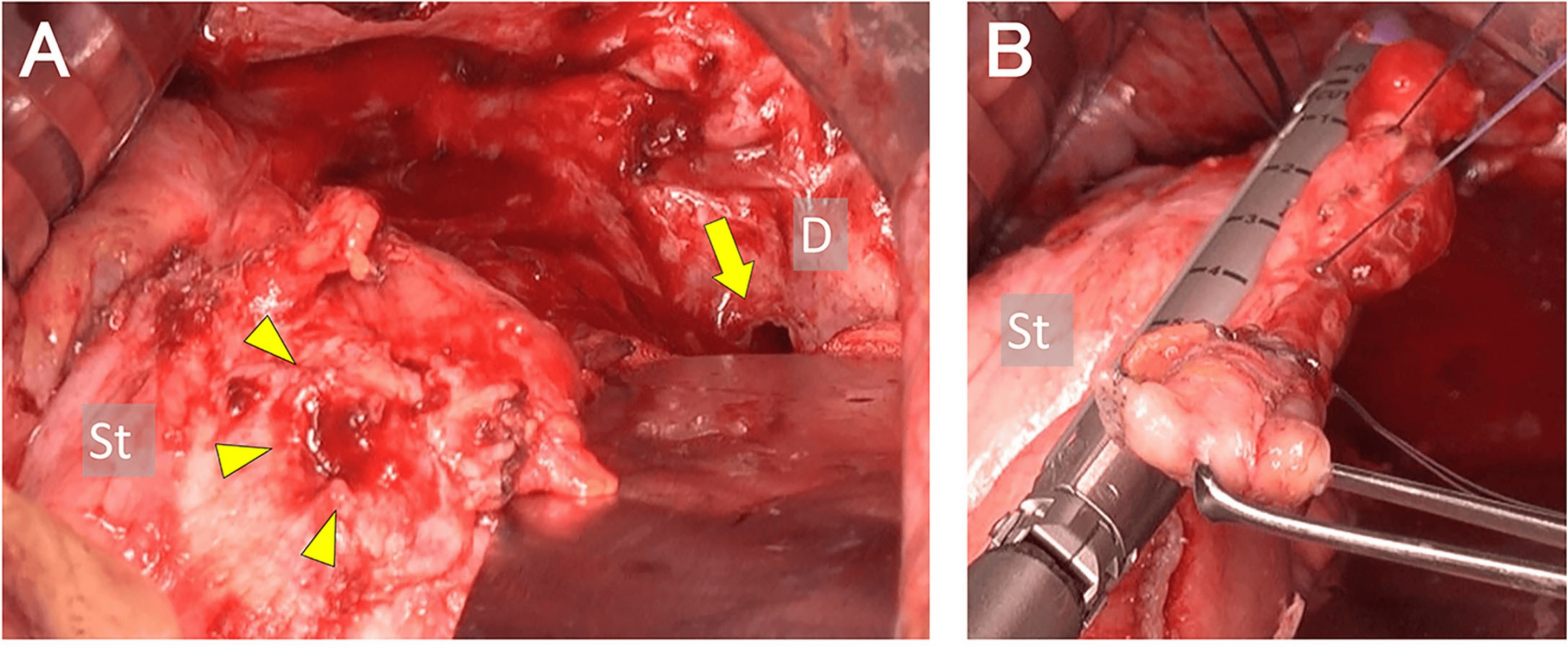
Intraoperative findings during laparotomy for the gastropleural fistula (GPF) (A) The fistula, measuring approximately 1.0 cm, located at the gastric fundus (arrowheads) and the left diaphragm (arrow) (B) Partial resection of the gastric fundus, including the hardened tissue surrounding the fistula. The gastric defect was repaired using Gambee sutures and autosuture device, linear staplers. St: stomach, D: diaphragm

During laparotomy, the GPF was identified, involving the gastric fundus to the left diaphragm, along with multiple peritoneal metastases. Adhesions in the left upper abdomen were lysed, revealing a 1.0 cm defect in the gastric fundus and left diaphragm (Figure 5A). The gastric fundus and surrounding hardened tissue were partially resected, and the gastric defect was repaired using Gambee sutures and linear staplers (Figure 5B).

The diaphragm defect was filled with the greater omentum. A jejunostomy tube was placed, and the left subdiaphragmatic space was drained. Histological examination of the resected gastric tissue confirmed poorly differentiated adenocarcinoma, consistent with the primary descending colon cancer.

Postoperatively, the patient was extubated, and EN via jejunostomy was initiated on postoperative day (POD) 2. The chest tube and left subdiaphragmatic drain were removed on POD 7 and POD 11, respectively. Oral intake was resumed on POD 9. There were no serious postoperative complications, however the patient’s nutritional status did not improve enough, performance status was reduced due to the progression of carcinomatous peritonitis, and the patient hoped for no further chemotherapy. The patient was discharged on POD 51 with a nasojejunal feeding tube for home medical care. Unfortunately, the patient passed away three months later. 

## Discussion

GPF is a rare and challenging condition to treat. Markowitz and Herter were the first to report cases of GPF, classifying the condition into three pathological categories: (I) perforation of the intrathoracic portion of the stomach within an esophageal hiatal hernia; (II) trauma; and (III) perforation of the stomach located entirely within the peritoneal cavity, followed by secondary abscess formation and erosion through the diaphragm into the pleural cavity [1]. Recent studies have proposed an additional category: (IV) direct perforation of the stomach into the pleural cavity without abscess formation [12]. The etiologies of GPF are diverse and include gastric peptic ulcers, malignancies, previous surgical procedures, and post-treatment complications following radiotherapy or chemotherapy [1-6].

Diagnosing GPF is challenging due to its rarity and variable clinical presentations, which may include fatigue, dyspnea, cough, fever, and pain in the left chest, back, or upper abdomen [2,3,8-9,11]. Radiological imaging plays a crucial role in diagnosis. Chest X-rays and CT scans often reveal hydropneumothorax or evidence of adhesion between the gastric fundus and the left diaphragm. Upper GI series, especially when combined with endoscopy, provide the most reliable diagnostic method by demonstrating contrast leakage from the stomach into the pleural cavity [7-9].

Management of GPF remains a topic of debate, largely depending on the underlying etiology and the patient’s overall condition. Conservative treatments, such as chest tube drainage, bowel rest, PN, and intravenous antibiotics, are typically the initial approach. While some patients achieve resolution with conservative measures alone, others require surgical intervention. Several invasive approaches, including laparotomy, thoracotomy, laparoscopy or endoscopic techniques such as clipping of the fistula or argon plasma coagulation, are generally effective [5-10]. However, these surgical interventions present challenges in cases of malignancy-associated GPF, which are associated with poorer outcomes [2,6,11,12].

A comprehensive PubMed search was conducted for studies published up to November 2024 using the keywords “gastropleural fistula,” “cancer,” and “chemotherapy.” Few reports of GPF associated with malignancy were identified. These cases can be categorized into the following etiologies: (a) Tumor rupture or invasion: Bulky tumors such as malignant lymphoma or metastases from renal cell carcinoma (RCC) or ovarian cancer, leading to communication between the gastric fundus and diaphragm [2,6,8,10,11]; (b) Gastric perforation: gastric ulcers or ischemia secondary to surgeries (e.g., lung cancer surgery), radiotherapy, or the use of steroids in chemotherapy for conditions such as metastatic RCC, head and neck cancer, or acute lymphoblastic leukemia [3,4,12,13]; (c) Infection: spread of infection, such as subdiaphragmatic abscess or empyema, arising from immunosuppression induced by chemotherapy for multiple myeloma or ovarian metastases [5,14]. Notably, no prior reports of GPF associated with colorectal cancer were identified in the literature.

A history of surgery involving left upper abdominal organs, such as splenectomy or left nephrectomy, may serve as a potential risk factor for GPF. There are limited reports in the literature linking GPF to a history of splenectomy or left nephrectomy, leading to the hypothesis that such procedures may alter the anatomical relationship between the gastric fundus and the diaphragm. Postoperative adhesion between these two structures could predispose to GPF [5,15]. Another hypothesis suggests that slight injuries to the stomach or diaphragm during emergent splenectomy, such as those performed for splenic abscesses or traumatic splenic laceration, may lead to late-onset complications like peptic ulcers or abscesses, potentially developing into GPF [16,17].

The optimal timing for surgical intervention in cases of GPF remains a topic of debate [5-8]. In this case, determining the timing of surgical management was particularly challenging, as the patient had recently undergone chemotherapy with anti-vascular endothelial growth factor (anti-VEGF) therapy. Surgical outcomes following anti-VEGF therapy can be compromised due to its adverse events, including delayed wound healing and GI perforation [18,19]. While there is no definitive consensus on the appropriate interval between the last administration of anti-VEGF therapy and surgery, some studies suggest that delaying the surgery for at least four weeks after the last bevacizumab administration is associated with a lower risk of complications [18-20]. Based on this evidence, a decision was made between the patient and primary physician to perform surgery four weeks after the patient’s last anti-VEGF treatment. On the other hand, we did not choose any endoscopic approaches, such as clipping or argon plasma coagulation, because the fistula could not be closed due to the surrounding hardened and fragile tissue by peritoneal metastases. Our approach was successful, with no postoperative wound healing complications, and the patient was able to resume oral intake for the remainder of her life.

In the present case, the GPF appears to be type 4, characterized by direct perforation between the gastric fundus and pleural cavity, and was likely caused by perforation at the site of a peritoneal metastasis during chemotherapy with anti-VEGF. To the best of our knowledge, this is the first reported case of GPF arising from peritoneal metastasis of colon cancer. Additionally, the patient’s history of splenectomy may have contributed to the outcome by promoting adhesion between the stomach and left diaphragm. It is conceivable that placing a physical barrier, such as omentum, between the gastric fundus and diaphragm during splenectomy could have reduced the risk of developing GPF.

## Conclusions

This is the first reported case of GPF associated with peritoneal metastasis of colon cancer. GPF is a rare and challenging complication in the treatment of malignant diseases, posing significant difficulties in diagnosis and management. Careful consideration of the timing of surgical interventions, guided by the patient’s overall condition and treatment history, is crucial for optimal outcomes. 
